# Follicular fluid biomarkers for human in vitro fertilization outcome: Proof of principle

**DOI:** 10.1186/s12953-016-0106-9

**Published:** 2016-11-14

**Authors:** Fang Chen, Carl Spiessens, Thomas D’Hooghe, Karen Peeraer, Sebastien Carpentier

**Affiliations:** 1 Leuven University Fertility Centre, UZ Leuven Campus Gasthuisberg, Herestraat 49, Leuven, Belgium; 2 Facility for Systems Biology based Mass Spectrometry (SYBIOMA), KU Leuven, Leuven, Belgium

**Keywords:** Peptide, Biomarker, Oocyte quality, Follicular fluid

## Abstract

**Background:**

Human follicular fluid (FF) is a unique biological fluid in which the oocyte develops in vivo, and presents an optimal source for non-invasive biochemical predictors. Oocyte quality directly influences the embryo development and hence, may be used as a predictor of embryo quality. Peptide profiling of FF and its potential use as a biomarker for oocyte quality has never been reported.

**Methods:**

This study screened FF for peptide biomarkers that predict the outcome of in vitro fertilization (IVF). Potential biomarkers were discovered by investigating 2 training datasets, consisting both of 17 samples and validating on an independent experiment containing 32 samples. Peptide profiles were acquired by nano-scale liquid chromatography coupled to tandem mass spectrometry (nano LC-MS/MS).

**Results:**

From the training datasets 53 peptides were found as potential biomarker candidates, predicting the fertilization outcome of 24 out of the 32 validation samples blindly (81.3% sensitivity, 68.8% specificity, AUC = 0.86). Seven potential biomarker peptides were identified. They were derived from: insulin-like growth factor binding protein-5, alpha-2-antiplasmin, complement component 3, inter-alpha-trypsin inhibitor heavy chain H1, serum albumin, protein diaphanous homolog 1 and plastin-3.

**Conclusions:**

The MS-based comprehensive peptidomic approach carried out in this study, established a novel panel of potential biomarkers that present a promising predictive accuracy rate in fertilization outcome, and indicates FF as an interesting biomarker resource to improve IVF clinic routine.

**Electronic supplementary material:**

The online version of this article (doi:10.1186/s12953-016-0106-9) contains supplementary material, which is available to authorized users.

## Background

Over the years, in vitro fertilization is associated with a high rate of multiple pregnancies, which presents both perinatal complications and economic complaints [1–3]. To reduce the incidence of multiple pregnancies, single embryo transfer (SET) is the only strategy [4, 5]. The selection of high quality embryos remains the major challenge in human assisted reproductive technology (ART). Worldwide, the selection of embryos has been based on morphological assessments. However, there is still a lack of evidence-based standard for ranking embryos and determining the embryo with the highest implantation potential [6]. The low rate of successful pregnancies creates the need to increase the predictive value for implantation.

Given the fact that RNA, proteins, and cellular machinery are provided by the oocyte during early zygote development, oocyte quality determines a big part of the embryo development and hence, may be used as a predictor of embryo quality [7]. Human follicular fluid (FF) has been attracting researchers’ interest since it is non-invasive and easily available. FF is a product of both the transfer of blood plasma constituents that cross the blood follicular barrier and of the secretory activity of granulosa and thecal cells [8]. FF is a complex mixture of proteins, metabolites, and ionic compounds, which have been found to reflect the stage of oocyte development and the degree of follicle maturation [9–14]. It has also been previously shown that altered FF composition is associated with a diminished reproductive capacity [14, 15]. Therefore, it is reasonable to think that some biochemical characteristics of the FF reflect oocyte quality and influence fertilization [16]. In body fluids, biomarkers are often low molecular weight peptides and proteins [17, 18]. Given the complexity of the numerous independent processes involved in oocyte maturation, it is unlikely that a single biomarker can classify the oocytes [19]. Application of powerful proteomic and peptidomic technologies in reproductive medical research may significantly contribute to help diagnosis but also the comprehensive understanding of reproductive processes. Several laboratories have demonstrated the feasibility of selecting peptide/protein diagnostic biomarkers in follicular fluid [20–22].

Efforts to explore the follicular fluid proteomic signature using different proteomic approaches have been carried out by several groups [19, 23–26]. The study performed by Spitzer et al. compared protein patterns in FF from immature and mature FF using two-dimensional gel electrophoresis (2-DE) [27], reporting considerable differences in protein patterns derived from fluids of immature compared with matured follicles. Hanrieder et al. coupled isoelectric focusing to nano liquid chromatography and MALDI TOF/TOF, and identified 69 proteins in FF of women undergoing IVF [28]. Twigt et al. using SDS-PAGE and isoelectric focusing followed by LC-MS/MS identified 246 FF proteins which are involved in acute phase response and immunological function [19]. Ambekar et al. combined three different methods of protein/peptide fractionation and identified 480 proteins in FF [24]. A more recent study performed by Zamah et al. identified 742 proteins in follicular fluid from fertile woman [22].

Despite decades of efforts, comparative studies on FF with respect to IVF outcome have not yet been done yet. Specific and sensitive proteomic biomarker candidates for IVF outcome have not been found. The low-molecular-weight (LMW) subset of proteome is termed the “peptidome”, including peptides and small proteins with molecular weights generally less than 10,000. At the early stage in the development of the peptidome field, the information content of peptides resides in two general categories: first, bioactive peptides and fragments shed from cells in the microenvironment, such as hormones and cytokines. Therefore, peptides may reflect the cell-to-cell communications taking place in the microenvironment [29]. The other is the cleavage products produced by enzymes and proteases as a consequence of certain physiological or pathological processes, such as apoptosis or necrosis, which may serve as reporters for biological enzymatic states of individuals [29–31]. Taking the diagnostic information carried by the peptidome into consideration, measuring panels of peptidome makers is expected to generate a higher level of prognostic capacity. Peptides are constantly generated in vivo by active synthesis, and by proteolytic processing of larger precursor proteins, often yielding protein fragments that mediate a variety of physiological functions [32]. This study aimed to reveal if peptide profiling of individual FF could become a new non-invasive predictive biomarker for oocyte quality, and attempted to discover candidate biomarkers for fertilization. In biomarker studies, candidate biomarkers need to be validated across a large number of samples because of normal clinical or biological variability. To ensure that the discovered biomarkers are truly associated with fertilization three experiments were designed with a relatively large population. We investigated the peptide profile of human follicular fluid with successful fertilization and unsuccessful fertilization from patients undergoing in vitro fertilization using LC_MS/MS. We additionally determined the protein identities of the discovered peptide biomarkers as a first step toward understanding the pathways in which they may function.

## Methods

### Study design

A total number of 66 follicular fluid samples from 50 couples undergoing IVF/ICSI treatment at the Leuven University Fertility Center were analyzed. All the patients were undergoing the first or second treatment cycle with a single embryo transferred (SET) and ranged from 18 to 36 years old. Patients with repeated implantation failures (cycle rank > 2) were not considered. Patients were fully informed and consents were obtained before oocyte retrieval. The study was approved by the Commission for Medical Ethics of the University Hospital Leuven (code ML6214).

The follicular fluid samples were analyzed in 3 experiments. The first training dataset contained 17 samples (8 successfully fertilized oocytes (showing 2 pronuclei), 9 unfertilized mature oocytes), the second training dataset contained 17 samples (7 successfully fertilized oocytes, 10 unfertilized mature oocytes). The validation dataset contained 32 samples (16 successfully fertilized oocytes, 16 unfertilized mature oocytes). All the fertilized oocytes in this study resulted in implantation after SET. Samples were randomly selected regardless the treatment of insemination (IVF/ICSI). Samples of training sets and validation sets were shown in Table 1. Patients’ characteristics were detailed in Table 2 and Additional file 1: Table S2.

**Table 1:** Characteristics and treatments of the training and validation cohorts

	Training	Validation
Characteristics	(*n* = 34)	(*n* = 32)
ICSI unfertilized mature oocytes(n)	11	7
ICSI fertilized oocytes (n)	10	7
IVF unfertilized mature oocytes(n)	8	9
IVF fertilized oocytes (n)	5	9

**Table 2:** Patients’ characteristics

	fertilized	non-fertilized
number of patients	31	35
maternal age	31.2 ± 2.7	31.7 ± 3.7
retrieved oocytes	10.6 ± 4.7	11.0 ± 4.3
matured oocytes	9.2 ± 3.6	9.6 ± 3.3
fertilized oocytes	6.2 ± 3.1	6.4 ± 2.8
IVF	14	17
ICSI	17	18
male factor	16	15
male factor and famale factor	4	6
anovulation	4	3
endometriosis	4	4
transport	2	1
unexplained	1	6

### Ovarian stimulation, oocyte retrieval and follicular fluid collection

The stimulation protocol used in this study has been published by Debrock [33]. Briefly, ovarian stimulation was carried out with gonadotropins (Menopur, Ferring, Copenhagen, Denmark; Gonal-F or Metrodin HP, Merck-Serono, Geneva, Switzerland; Puregon, Organon, Oss, The Netherlands) and GnRH agonists (Buserlin acetate, Suprefact; Hoechst, Frankfurt, Germany) during a long or short protocol. The follicular response was monitored serum oestradiol levels and transvaginal ultrasound measurements. The hCG, 10,000 IU, was administered when at least three follicles reached a diameter of 17 mm. Oocyte retrieval was performed 35 h after hCG injection by ultrasound guided transvaginal aspiration. The luteal phase was supported with intravaginal application of P (600 mg/day, Utrogestan; Besins, Drogenbos, Belgium) started at the evening of the hCG injection.

Follicular fluid was aspirated and collected separately, then kept on ice immediately. Each follicle was flushed twice. To maintain a stable pH in a room atmosphere condition, commercial medium Dulbecco’s phosphate-buffered saline (DPBS; Gibco, Paisley, UK) was used as flushing medium. For each FF sample, the volume and color appearance (yellow, light reddish, reddish, dark reddish and red) were recorded. Only FF of yellow or light reddish was allowed for further analysis.

The samples were centrifuged at 1500 * g for 10 min. The supernatant was transferred to a cryotube and stored in liquid nitrogen until further processing.

### In vitro insemination/Intracytoplasmic sperm injection

Prior to fertilization, oocytes were washed 4 times with wash medium GM501 (GM 501 Wash, Gynemed Lensahn, Germany) after retrieval in order to minimize the amount of blood/follicular fluid. Oocytes were placed separately in a 4-well dish (Nunc, Thermo Fisher Scientific, Kamstrupvej, Denmark) containing wash medium GM501 (GM 501 Wash, Gynemed Lensahn, Germany) under oil. Spermatozoa for the IVF/ICSI procedure were prepared using standard density gradient procedures (Isolate, Irvine Scientific, USA) or, in cases with very low sperm quality, diluted and centrifuged twice at 300 g for 10 min. Standard IVF/ICSI procedures were performed 2–6 h after oocyte retrieval. In the IVF procedure, oocytes were inseminated with 10,000 progressively motile spermatozoa per oocyte. In the ICSI procedure, the cumulus and corona cells were removed with hyaluronidase (conc.80 IU/m, Gynemed, Lensahn, Germany). The oocytes were injected with single sperm in a 20 μl droplet of medium. The injected oocytes were cultured individually in 20 μl culture medium (GM 501 Culture, Gynemed Lensahn, Germany) droplets under oil. On Day 1 (16–20 h after insemination/injection) fertilization was evaluated.

### Peptide extraction

Follicular fluid samples (500 μl) were transferred to extraction tubes (2 ml) and mixed with an equal amount of lysis buffer containing 30 mM Tris (SIGMA, St. Louis, USA), 8 M urea (Acros Organics, New Jersey, USA), 5 mM Dithiothreitol DTT, Applichem, Darmstadt, Germany). They were vortexed thoroughly and centrifuged at 13,000 rpm for 10 min at room temperature. The supernatant was then transferred to a 3 K Da filter Microcon YM-30 filters (Millipore, Billerica, MA, USA), the filter devices were subsequently centrifuged at 14,000 g for 30 min and the flow through was collected. Iodoacetamide (IAA, SIGMA, St. Louis, USA) was added to a final concentration of 0.015 M and the samples were incubated for 30 min at room temperature in dark. Then trifluoroacetic acid (TFA) was added to a final concentration of 0.1%. The mixture was cleaned via solid phase extraction (SPE) using Pepclean C18 spin column (Thermo Scientific) according to the manufactuer’s instructions and eluted in a final volume of 40 μL. Subsequently, the samples were dried in a vacuum operator. The dried sample was kept at −20°C until analysis. The peptides were dissolved in 10 μl of 0.1% formic acid (FA) and 5% acetonitrile (ACN).

### MS Data Processing

Five microliters from each sample were injected and separated on an Ultimate® 3000 RSLCnano system (Dionex, Thermo Scientific, Netherlands) equipped with a Thermo Scientific™ Acclaim™ PepMap™ RSLC Nano-Trap Column with nanoViper™ Fittings, 3 μm Particle Size. The samples were separated using a buffer A (water/0.1% FA) and buffer B (water 20%/ACN 80%/FA 0.08%) and a Thermo Scientific™ EASY-Spray™ column PepMap™ RSLC, C18, 2 μm, 100 Å, 50 μm x 150 mm using a gradient of 4 to 10% B in 12 min followed by a gradient of 10 to 35% B in 20 min, a gradient 35 to 65% B in 5 min and then a final elution and re-equilibration step at 95 and 5% buffer B respectively for 9 min. The flow-rate was set at 0.300 μL/min. The hybrid quadrupole orbitrap mass spectrometer, Q Exactive (Thermo Scientific), was operated in positive ion mode with a nanospray voltage of 1.5 kV and a source temperature of 250°C. ProteoMass LTQ/FT-Hybrid ESI Pos. Mode CalMix (MSCAL5-1EA SUPELCO, Sigma-Aldrich) was used as an external calibrant and the lock mass 445.12003 as an internal calibrant. The instrument was operated in data-dependent acquisition (DDA) mode with a survey MS scan at a resolution of 70,000 for the mass range of m/z 400–1600 for precursor ions, followed by MS/MS scans of the top 10 most intense peaks with +2, +3, +4 and +5 charged ions above a threshold ion count of 16,000 at 17,500 resolution using normalized collision energy (NCE) of 25 eV with an isolation window of 3.0 m/z, an apex trigger 5–15 s and a dynamic exclusion of 10 s. All data were acquired with Xcalibur 3.0 software (Thermo Scientific).

The LC-MS data were imported to Progenesis Nonlinear software Progenesis v4.1 (Nonlinear Dynamics, UK) for alignment and normalization to compare the different sample runs. The software selected automatically the sample run with the greatest similarity to all other runs as the reference alignment. The aligned runs containing all ion peak information from all sample files was exported as mgf and send to Mascot (version 2.2.06; database swissprot 15,720 accessions). Mass tolerance was set to 10 ppm for MS and 20 mmu for MS/MS, and no cleavage enzyme for protein digestion was chosen. Search parameters allowed for carbamidomethylation as fixed modification and oxidation of M as variable modification. Search results were evaluated by Scaffold™ (released version 4.4.5) combining mascot and X! Tandem. Only peptides with an expected value of <0.05 are reported.

### Statistical analysis

The aligned and normalized peptide data were exported from Progenesis as csv format. The determination of candidate peptides in the training datasets was accomplished by partial least square discriminative analysis (PLS-DA) using the NIPALS algorithm of Statistica 8.1 (Statsoft). The analysis of variance (ANOVA) was taken over from Progenesis. The prediction capability of biomarker candidates and blind classifying was evaluated by principle component analysis (PCA) using the NIPALS algorithm of Statistica 8.1 (Statsoft).

To evaluate the predictive capability of the biomarker candidates, receiver operating characteristic (ROC) analysis was carried out with MATLAB (2014 a).

Probability calculations were performed according to the binomial distribution. The probability of getting exactly x successes in n trials is given by the probability mass function: b(x; n, P) = nCx * P^x^ * (1 - P)^n – x^.

## Results

### Training datasets

**Fig. 1:**
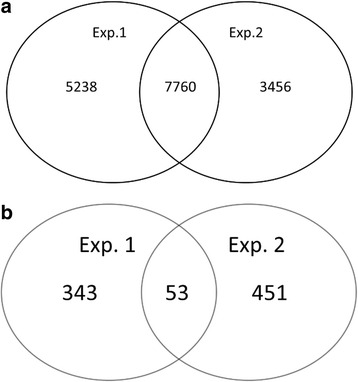
Venn diagram of peptides between two training experiments. **a** detected peptides; **b** important peptides to fertilization

**Fig. 2:**
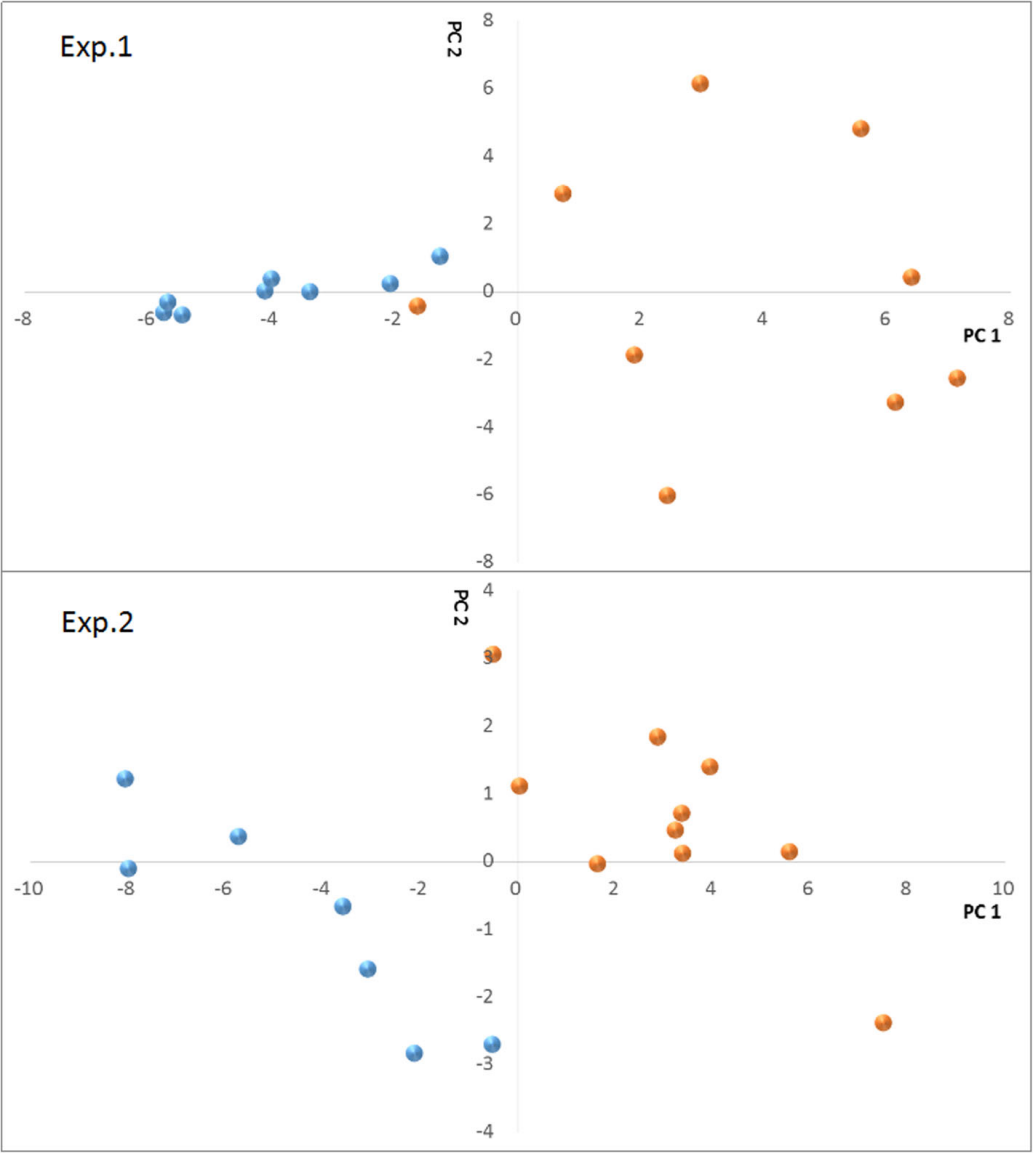
Screening candidate biomarkers in FF for fertilization in training cohorts. PLS scores show evident clustering between fertilized (*blue*) and non-fertilized (*orange*) samples. Samples were separated well in the first component

**Fig. 3:**
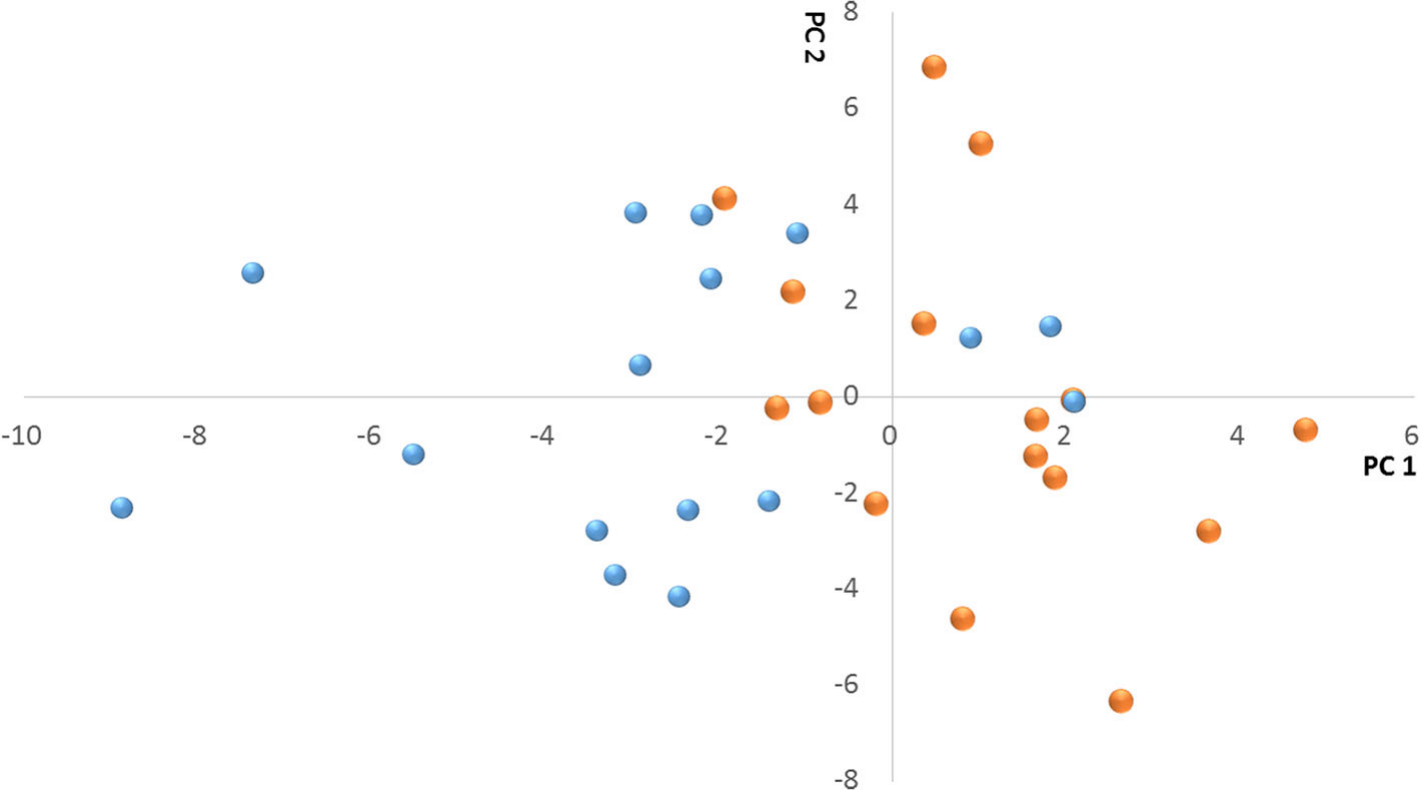
ROC curve test of FF peptide biomarker candidates. *Blue*: ROC curve on training dataset; *Red*: ROC curve on validation dataset (*p*-value =0.13)

To avoid over fitting of the PLS-DA model, we firstly examined 2 training datasets of 17 samples and evaluated the differences at the peptidome level of the individual follicular fluid samples. Peptides that met both standards: (1) *p* < 0.1 with ANOVA, and (2) top 10% on variable importance ranking were selected and compared for each experiment. In the first experiment, 12,998 peptides were detected. 394 peptides met the criteria. In the second experiment, 11,216 peptides were detected, of which 7760 peptides were in common with the first experiment (Fig. 1a), and 504 peptides met the criteria. Among all the interesting candidate peptides described above, 53 were common to both training datasets (Fig. 1b). Results of partial least square discriminate analysis were shown in Fig. 2. Those peptides are listed in Table 3. Of the 53 peptides, 9 peptides were upregulated in the fertilized group and 44 were upregulated in the non-fertilized group (Table 3). The area under ROC curve (AUC) was 0.97, with the sensitivity of 0.82 and the specificity of 0.84 (Fig. 3).

**Table 3:** Peptides set as biomarkers differentially detected between fertilized group and non-fertilized group

m/z	RT	Charge	Highest mean condition	max fold change		Protein	Accession number	Peptide sequence
				Exp.1	Exp.2			
401.22 ± 0.00	28.2 ± 0.11	2*	non-fertilized	8.94^b^	3.07^c^	ALBU_Human	P02768	AASQAALGL
402.23 ± 0.00	25.3 ± 0.66	1	non-fertilized	2.16^b^	2.44^c^			
409.18 ± 0.00	24.8 ± 0.22	1	non-fertilized	2.7^a^	1.99^b^			
410.17 ± 0.00	36.4 ± 0.26	1	fertilized	3.09^a^	11.07^b^			
412.34 ± 0.00	44.3 ± 0.02	1*	non-fertilized	3.36^b^	2.78^b^			
414.34 ± 0.01	43.3 ± 2.37	1*	non-fertilized	1.93^c^	2.00^c^			
416.37 ± 0.00	46.0 ± 0.15	1*	non-fertilized	2.11^c^	14.22^a^			
424.29 ± 0.01	46.5 ± 0.15	1*	fertilized	4.18^b^	2.24^a^			
426.32 ± 0.02	48.0 ± 0.77	1	fertilized	1.49^c^	2.33^b^			
438.36 ± 0.02	52.4 ± 1.14	1	non-fertilized	2.67^c^	2.76^a^			
439.28 ± 0.00	25.0 ± 0.38	1*	non-fertilized	2.88^b^	215.28^b^			
442.94 ± 0.00	27.2 ± 0.60	3	non-fertilized	21.09^b^	2.57^a^			
459.72 ± 0.00	22.1 ± 0.30	2*	non-fertilized	27.37^b^	12.84^b^			
475.01 ± 0.00	23.4 ± 0.48	4*	non-fertilized	5.63^b^	1.77^b^			
477.28 ± 0.00	47.6 ± 0.48	1*	non-fertilized	25.53^a^	25.84^c^			
478.62 ± 0.00	41.8 ± 0.00	3*	non-fertilized	3.02^b^	1.18^c^			
480.80 ± 0.00	40.5 ± 0.82	2*	fertilized	1.22^c^	1.30^c^			
490.24 ± 0.02	46.5 ± 0.25	1	fertilized	1.70^b^	8.68^c^			
494.29 ± 0.02	46.4 ± 0.97	1*	non-fertilized	549.84^a^	2.59^b^	IGBP-5_Human	P24593	FVGGAENTAHPRII
496.97 ± 0.00	43.1 ± 0.39	3	fertilized	1.36^b^	1.40^b^			
500.24 ± 0.00	26.7 ± 0.26	3	non-fertilized	341.3^c^	2.55^c^			
526.41 ± 0.02	52.1 ± 1.08	1	non-fertilized	3.59^b^	3.10^a^			
528.28 ± 0.00	34.1 ± 0.11	2*	fertilized	3.61^b^	1.94^c^	CO3_Huamn	P01024	IHWESASLL
530.29 ± 0.00	45.8 ± 1.13	1*	fertilized	1.87^c^	6.26^c^			
539.66 ± 0.00	44.7 ± 0.41	6	non-fertilized	2.08^b^	1.73^b^			
545.33 ± 0.00	44.8 ± 0.26	6	non-fertilized	Infinit^a^	2.08^b^			
546.99 ± 0.00	44.7 ± 0.42	6	non-fertilized	1.93^b^	1.60^b^			
552.30 ± 0.00	28.7 ± 0.11	5	non-fertilized	68.46^c^	2.24^b^			
552.67 ± 0.00	44.7 ± 0.40	6	non-fertilized	3.05^c^	1.60^c^			
554.33 ± 0.00	44.7 ± 0.41	6	non-fertilized	2.00^c^	1.70^b^			
558.40 ± 0.00	51.1 ± 0.31	2	non-fertilized	1795.51^a^	Infinit^a^			
570.43 ± 0.02	51.9 ± 1.06	1	non-fertilized	4.31^b^	3.77^c^			
574.68 ± 0.00	44.8 ± 0.40	6	non-fertilized	4.33^b^	2.39^c^			
576.35 ± 0.00	44.8 ± 0.41	6	non-fertilized	2.24^b^	1.94^b^			
577.28 ± 0.00	33.5 ± 0.77	1*	non-fertilized	1.42^b^	2.26^b^			
579.18 ± 0.00	44.8 ± 0.39	6	non-fertilized	2.19^b^	1.95^c^			
588.43 ± 0.01	45.5 ± 1.66	1	non-fertilized	43.29^b^	4.46^c^			
594.30 ± 0.00	24.0 ± 0.14	2*	fertilized	3.09^b^	2.90^a^	ITIH1_human	P19827	LPDRVTGVDTD
602.42 ± 0.00	51.4 ± 1.02	2	non-fertilized	164.28^c^	Infinit^a^			
609.72 ± 0.00	30.0 ± 0.00	5	non-fertilized	10.0^b^	1.91^a^			
643.33 ± 0.00	29.0 ± 1.59	2*	non-fertilized	Infinit^c^	2.59^a^	A2AP_human	P08697	MEPLGRQLTSGP
658.49 ± 0.02	51.7 ± 0.99	1	non-fertilized	7.59^b^	11.57^b^			
702.51 ± 0.02	51.6 ± 1.01	1	non-fertilized	6.36^b^	12.66^c^			
703.75 ± 0.00	31.1 ± 1.97	5	non-fertilized	Infinit^a^	1.79^b^			
727.59 ± 0.00	28.0 ± 0.07	4*	non-fertilized	1266.81^a^	2.03^b^			
740.96 ± 0.00	30.6 ± 0.05	5	non-fertilized	4.35^b^	1.91^a^	PLST_HUMAN	P13797	DGETLEELMKLSPEELLLRWANFHLENSGWQ
755.34 ± 0	12.2 ± 0.36	2	non-fertilized	2.04^b^	2.16^a^			
761.97 ± 0.00	31.0 ± 0.01	5	non-fertilized	6.85^b^	2.10^a^			
805.39 ± 0.00	35.8 ± 0.01	1*	non-fertilized	Infinit^c^	7.38^b^			
859.44 ± 0.00	29.4 ± 0.04	4	non-fertilized	Infinit^a^	2.94^a^			
869.68 ± 0.00	32.7 ± 0.15	4	non-fertilized	Infinit^b^	3.92^c^			
924.74 ± 0.00	38.0 ± 0.41	3*	non-fertilized	Infinit^c^	9.77^b^			
953.51 ± 0.00	29.1 ± 0.23	3	non-fertilized	12.72^b^	3.24^c^	DIAP1_HUMAN	O60610	AEPHFLSILQHLLLVRNDYEARPQ

^a^ *p* < 0.01; ^b^ *p* < 0.05; ^c^ *p* < 0.1

*:significant difference in validation experiment

### External validation

**Fig. 4:**
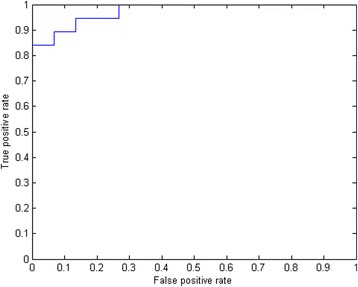
Visual identification of the hFF samples using the 53 peptide biomarker candidates. PCA score plots derived from PCA of 53 important biomarker candidates of 32 hFF with fertilized oocytes or non-fertilized oocytes. PC1, explaining 36.6% of the variability is clearly associated to the outcome of fertilization

The most reliable way of biomarker discovery is to test the candidates with a large cohort of samples. From the perspective of generality, 32 samples from an external study population were analyzed and we predicted the oocyte fertilization outcome blindly via principle component analysis (PCA). The 53 potential biomarkers discriminate 24 out of the 32 candidates (Fig. 4). The chance of discriminating 24/32 cases randomly is 0.25% according to the binomial distribution. As indicated above, 44/53 markers are negatively correlated to fertilization.

Receiver operation characteristic (ROC) analysis indicates that the 53 peptides panel has a high predictive ability to differentiate FF samples upon fertilization outcome, with the AUC value of 0.86, the sensitivity of 81.3%, and the specificity of 68.8%.

### Peptide identification

The identification of non-tryptic peptides is challenging due to the absence of a known cleavage side and the absence of a basic amino acid at the C-terminus. In total 7102 unique peptides were identified, belonging to 159 proteins (protein false discovery rate (FDR) 0.06% and peptide FDR 0.00% (Additional file 2: Table S1 and Additional file 3: Table S3)). From the 53 peptides panel, we were able to identify 7 peptides derived from 7 different proteins (Table 3). The MS spectrum of the identified peptides is displayed in Additional file 4: Figure S1.

### Subcellular and functional annotation of identified peptides

We checked the subcellular localization of all the peptides identified. We found that 5 proteins were localized at extracellular space. Insulin-like growth factor binding protein-5 (IGBP-5) is secreted by granulosa cells, four proteins are predicted to come from the circulation system. Two proteins were predicted to be localized in the intracellular region, cytoplasm and membrane.

## Discussion

The morphological assessments for human embryo selection in clinic IVF routine is not fully satisfying. In the last decades, scientists have been attempting to improve the embryo selection. As the microenvironment of oocyte in vivo, the protein/peptide content of human follicular fluid has attracted researchers’ interest. However, to date, research correlates protein/peptide profiling to IVF outcome has been done yet. The present study focused on the peptide profile of follicular fluid with different fertilization outcomes to screen potential peptide biomarkers for fertilization. Our results have shown altered level of certain peptides contributed to the fertilization outcome. By external validation, we confirmed the classification capability of these peptides. We think that the findings reported identify a pool of peptides from which novel IVF-related biomarkers could be discovered.

In our study, the concentration of one peptide (m/z = 401.23) was significantly less abundant in the fertilized group and was identified as a fragment derived from serum albumin (ALBU_Human) (Table 3). In literature, albumin as a protein has been positively correlated to oocyte quality. Junko et al. [34] proposed that the biochemically reduced state of albumin in FF may play an important role in protecting oocytes from oxidative damage. Our data point towards a negative correlation at the fragment peptide level and might be related to proteolytic processing.

We identified m/z = 494.59 derived from insulin-like growth factor binding protein-5 (IBP-5_Human) as a second interesting negative biomarker (Table 3). IGF binding proteins (IGFBP) inhibit insulin-like growth factor (IGF) actions. The IGF system plays an important role in regulating ovarian follicular development and steroidogenesis [35] and IGFBP proteolysis is a major mechanism for regulating IGF bioavailability [36]. The gene of IGFBP-5 was reported to be highly expressed in rat primary and secondary follicles while dominant follicles were devoid of IGFBP-5 mRNA [37]. In several other studies [38–42], dominant follicles were characterized by decreased levels of low molecular weight IGF-binding proteins (IGF-2,4, and −5). The high abundance of peptide derived from IGFBP-5 may reflect the increased level of IGFBP-5 in preovulatory follicle, which reduces the activity of growth factors, or by an increased activity of its protease or both.

We identified m/z = 643.33 derived from Alpha-2-antiplasmin (A2AP_Human) as a third interesting negative biomarker (Table 3). Alpha-2-antiplasmin is a serine protease inhibitor, involved in negative regulation of plasminogen activation. Bayasula et al. confirmed the presence of A2AP in human FF [43]. The major targets of this inhibitor are plasmin. Plasmin activity is believed to be involved in physiological processes such as ovulation [44], cumulus cell layer expansion [44, 45], oocyte maturation [44, 46] and fertilization [47, 48]. Hormone-induced coordinated expression of tissue-type PA (tPA) produced mainly by granulosa cells in the prevolulatory follicles is responsible for a controlled and directed proteolysis leading to the rupture of follicles [45]. The urokinase-type PA (uPA), activates latent proteinases or growth factors, playing an essential role in the early growing follicles during cell proliferation and migration [49]. In addition, plasmin could active pro-enzymes of the matrix metalloproteinase, which in turn, may also be involved in ovarian function and regulate follicular development [49, 50]. In sheep and rat, intra-follicular injection of A2AP suppresses ovulation of preovulatory follicles [51, 52]. Huarte et al. [48] reported that the addition of plasminogen to mouse IVF medium increased the yield of fertilized eggs, while the addition of plasmin inhibitors resulted in a significant decrease. Here, for the first time, we indicate that the differential status of A2AP may be associated with immature oocyte developmental stage and might explain the negative biomarker.

We identified m/z = 528.28 as a peptides derived from Complement component 3 (CO3_Human) in hFF (Table 3) to be present at a significantly higher level in fertilized FF group in our study. CO3 has been specifically located in ovarian follicular fluid in early 1990s [53], however, the roles of CO3 and its derivatives in FF are not yet fully known, and the association between CO3 and reproductive capability remains unclear. Gonzales et al. and Hashemitabar et al. found significantly higher concentrations of the CO3 complement in the FF of fertilized oocytes [11, 20]. In porc [54], a derivative of CO3, iCO3b was identified to positively influence oocyte maturation. Those studies are consistent with our finding as a positive biomarker. In contrast, a study assessing the FF of IVF patients showed decreased expression of complement CO3 in their fertilized group [25]. The major difference of Estes’ study was the inclusion of the oocyte number as dependent variable. Instead of predicting the outcome of fertilization, it might predict the differences between good and poor responders. Additional studies are needed to establish the actual influence of the complement cascade on reproductive aging and IVF outcome.

We identified m/z = 594.29 as derived from Inter-alpha-trypsin inhibitor heavy chain H1 (ITIH1_Human) as a positive biomarker (Table 3). Proteins of the inter-alpha-trypsin inhibitor family have been identified as a serum factor responsible for the stabilization of the expanding cumulus mass [55]. These proteins do not enter the follicle until it responds to an ovulatory stimulus. The luteinizing hormone (LH) surge increases the permeability of the charge and size-selective blood follicle barrier, allowing these serum glycoproteins to enter the antral cavities of responding follicles [56, 57]. Once within the antral cavity, the inter-alpha-trypsin inhibitor (ITI) covalently crosslinked to hyaluronan (HA), driving conformational changes and thereby remodeling the morphology and physicochemical properties of HA-rich extracellular matrices. This modification is the only naturally occurring covalent modification of HA known to date. The interaction was shown to be critical to matrix stabilization in expansion of the cumulus-oocyte complex (COC) [58, 59]. Huang et al. have shown that HA interacts strongly with ITIH1 and ITIH2 in vitro, and this binding was highly resistant to ionic strength and pH [60]. In another vivo study, mice lacking intact IαI family members fail to form a stable cumulus matrix and the naked ovulated oocytes are not fertilized in vivo [58].

## Conclusion

Despite progress in the treatment of IVF and advances in novel techniques, selection of embryos based on the morphologic and morphometric parameters alone is not fully satisfactory. More accurate selection of oocytes and embryos should improve success rates after IVF treatment. Follicle development and oocyte maturation, require or bring about changes to oocyte microenvironment. Given the complexity of the numerous independent processes involved in oocyte development, it is unlikely that a single biomarker can predict the result of in vitro fertilization. By characterization of the FF peptidome, we present a profile of biomarkers associated with fertilization outcome. This may offer prognostic information aiding the selection of the most viable oocytes and hence embryos. The current results confirmed our hypothesis that peptide profiling is a promising approach to screen biomarkers for fertilization potential. Comparison of our results with proteomic studies of hFF indicates that the different analytical tools each bring their own selectivity.

## Additional files

Additional file 1: Table S2. Patients’ characteristics. (XLSX 12 kb)
Additional file 2: Table S1. Ne protein identifications. (XLSX 17 kb)
Additional file 3: Table S3. Spectrum report. (XLSX 17 kb)
Additional file 4: Figure S1. MS spectrum form of identified peptides. (TIF 2099 kb)

## Abbreviations

A2AP: Alpha-2-antiplasmin
ART: Assisted reproductive technology
CO3: Complement component 3
FDR: False discovery rate (FDR)
FF: Follicular fluid
IGBP-5: Insulin-like growth factor binding protein-5
ITIH1: Inter-alpha-trypsin inhibitor heavy chain H1
LC-MS/MS: Liquid chromatography-tandem mass spectrometry
LMW: Low-molecular-weight
MS: Mass spectrometry
PCA: Principle component analysis
PLS: Partial least square
SET: Single embryo transferred (SET)

## References

[1] Fauser BC, Devroey P, Macklon NS (2005). Multiple birth resulting from ovarian stimulation for subfertility treatment. Lancet.

[2] Gerris JM (2005). Single embryo transfer and IVF/ICSI outcome: a balanced appraisal. Hum Reprod Update.

[3] Makhseed M, Al-Sharhan M, Egbase P, Al-Essa M, Grudzinskas JG (1998). Maternal and perinatal outcomes of multiple pregnancy following IVF-ET. Int J Gynaecol Obstet.

[4] Pandian Z, Bhattacharya S, Ozturk O, Serour G, Templeton A. Number of embryos for transfer following in-vitro fertilisation or intra-cytoplasmic sperm injection. Cochrane Database Syst Rev. 2009;7:997–1005. 10.1002/14651858.CD003416.pub3 19370588

[5] Thurin A, Hausken J, Hillensjo T, Jablonowska B, Pinborg A, Strandell A, Bergh C (2004). Elective single-embryo transfer versus double-embryo transfer in in vitro fertilization. N Engl J Med.

[6] Kovalevsky G, Patrizio P (2005). High rates of embryo wastage with use of assisted reproductive technology: a look at the trends between 1995 and 2001 in the United States. Fertil Steril.

[7] Sirard MA, Richard F, Blondin P, Robert C (2006). Contribution of the oocyte to embryo quality. Theriogenology.

[8] Fortune JE (1994). Ovarian follicular growth and development in mammals. Biol Reprod.

[9] Appasamy M, Jauniaux E, Serhal P, Al-Qahtani A, Groome NP, Muttukrishna S (2008). Evaluation of the relationship between follicular fluid oxidative stress, ovarian hormones, and response to gonadotropin stimulation. Fertil Steril.

[10] Fahiminiya S, Reynaud K, Labas V, Batard S, Chastant-Maillard S, Gerard N (2010). Steroid hormones content and proteomic analysis of canine follicular fluid during the preovulatory period. Reprod Biol Endocrinol.

[11] Hashemitabar M, Bahmanzadeh M, Mostafaie A, Orazizadeh M, Farimani M, Nikbakht R (2014). A proteomic analysis of human follicular fluid: comparison between younger and older women with normal FSH levels. Int J Mol Sci.

[12] Mason HD, Willis DS, Beard RW, Winston RM, Margara R, Franks S (1994). Estradiol production by granulosa cells of normal and polycystic ovaries: relationship to menstrual cycle history and concentrations of gonadotropins and sex steroids in follicular fluid. J Clin Endocrinol Metab.

[13] Monteleone P, Giovanni Artini P, Simi G, Casarosa E, Cela V, Genazzani AR (2008). Follicular fluid VEGF levels directly correlate with perifollicular blood flow in normoresponder patients undergoing IVF. J Assist Reprod Genet.

[14] Ocal P, Aydin S, Cepni I, Idil S, Idil M, Uzun H, Benian A (2004). Follicular fluid concentrations of vascular endothelial growth factor, inhibin A and inhibin B in IVF cycles: are they markers for ovarian response and pregnancy outcome?. Eur J Obstet Gynecol Reprod Biol.

[15] Wu YT, Wang TT, Chen XJ, Zhu XM, Dong MY, Sheng JZ, Xu CM, Huang HF (2012). Bone morphogenetic protein-15 in follicle fluid combined with age may differentiate between successful and unsuccessful poor ovarian responders. Reprod Biol Endocrinol.

[16] Revelli A, Delle Piane L, Casano S, Molinari E, Massobrio M, Rinaudo P (2009). Follicular fluid content and oocyte quality: from single biochemical markers to metabolomics. Reprod Biol Endocrinol.

[17] Rodthongkum N, Ramireddy R, Thayumanavan S, Richard WV (2012). Selective enrichment and sensitive detection of peptide and protein biomarkers in human serum using polymeric reverse micelles and MALDI-MS. Analyst.

[18] Luchini A, Fredolini C, Espina BH, Meani F, Reeder A, Rucker S, Petricoin EF, Liotta LA (2010). Nanoparticle technology: addressing the fundamental roadblocks to protein biomarker discovery. Curr Mol Med.

[19] Twigt J, Steegers-Theunissen RP, Bezstarosti K, Demmers JA (2012). Proteomic analysis of the microenvironment of developing oocytes. Proteomics.

[20] Gonzales J, Lesourd S, Van Dreden P, Richard P, Lefebvre G, Vauthier Brouzes D (1992). Protein composition of follicular fluid and oocyte cleavage occurrence in in vitro fertilization (IVF). J Assist Reprod Genet.

[21] Wunder DM, Mueller MD, Birkhauser MH, Bersinger NA (2005). Steroids and protein markers in the follicular fluid as indicators of oocyte quality in patients with and without endometriosis. J Assist Reprod Genet.

[22] Zamah AM, Hassis ME, Albertolle ME, Williams KE (2015). Proteomic analysis of human follicular fluid from fertile women. Clin Proteomics.

[23] Angelucci S, Ciavardelli D, Di Giuseppe F, Eleuterio E, Sulpizio M, Tiboni GM, Giampietro F, Palumbo P, Di Ilio C (1764). Proteome analysis of human follicular fluid. Biochim Biophys Acta.

[24] Ambekar AS, Nirujogi RS, Srikanth SM, Chavan S, Kelkar DS, Hinduja I, Zaveri K, Prasad TS, Harsha HC, Pandey A, Mukherjee S (2013). Proteomic analysis of human follicular fluid: a new perspective towards understanding folliculogenesis. J Proteomics.

[25] Estes SJ, Ye B, Qiu W, Cramer D, Hornstein MD, Missmer SA (2009). A proteomic analysis of IVF follicular fluid in women < or = 32 years old. Fertil Steril.

[26] Liu AX, Zhu YM, Luo Q, Wu YT, Gao HJ, Zhu XM, Xu CM, Huang HF (1770). Specific peptide patterns of follicular fluids at different growth stages analyzed by matrix-assisted laser desorption/ionization time-of-flight mass spectrometry. Biochim Biophys Acta.

[27] Spitzer D, Murach KF, Lottspeich F, Staudach A, Illmensee K (1996). Different protein patterns derived from follicular fluid of mature and immature human follicles. Hum Reprod.

[28] Hanrieder J, Nyakas A, Naessen T, Bergquist J (2008). Proteomic analysis of human follicular fluid using an alternative bottom-up approach. J Proteome Res.

[29] Liotta LA, Petricoin EF (2006). Serum peptidome for cancer detection: spinning biologic trash into diagnostic gold. J Clin Invest.

[30] Li B, Predel R, Neupert S, Hauser F, Tanaka Y, Cazzamali G, Williamson M, Arakane Y, Verleyen P, Schoofs L (2008). Genomics, transcriptomics, and peptidomics of neuropeptides and protein hormones in the red flour beetle Tribolium castaneum. Genome Res.

[31] Petricoin EF, Belluco C, Araujo RP, Liotta LA (2006). The blood peptidome: a higher dimension of information content for cancer biomarker discovery. Nat Rev Cancer.

[32] Lai ZW, Petrera A, Schilling O (2015). The emerging role of the peptidome in biomarker discovery and degradome profiling. Biol Chem.

[33] Debrock S, Melotte C, Spiessens C, Peeraer K, Vanneste E, Meeuwis L, Meuleman C, Frijns JP, Vermeesch JR, D’Hooghe TM (2010). Preimplantation genetic screening for aneuploidy of embryos after in vitro fertilization in women aged at least 35 years: a prospective randomized trial. Fertil Steril.

[34] Otsuki J, Nagai Y, Matsuyama Y, Terada T, Era S (2012). The influence of the redox state of follicular fluid albumin on the viability of aspirated human oocytes. Syst Biol Reprod Med.

[35] Thierry van Dessel HJ, Chandrasekher Y, Yap OW, Lee PD, Hintz RL, Faessen GH, Braat DD, Fauser BC, Giudice LC (1996). Serum and follicular fluid levels of insulin-like growth factor I (IGF-I), IGF-II, and IGF-binding protein-1 and −3 during the normal menstrual cycle. J Clin Endocrinol Metab.

[36] Nyegaard M, Overgaard MT, Su YQ, Hamilton AE, Kwintkiewicz J, Hsieh M, Nayak NR, Conti M, Conover CA, Giudice LC (2010). Lack of functional pregnancy-associated plasma protein-A (PAPPA) compromises mouse ovarian steroidogenesis and female fertility. Biol Reprod.

[37] Erickson GF, Nakatani A, Ling N, Shimasaki S (1992). Localization of insulin-like growth factor-binding protein-5 messenger ribonucleic acid in rat ovaries during the estrous cycle. Endocrinology.

[38] Rivera GM, Fortune JE (2003). Selection of the dominant follicle and insulin-like growth factor (IGF)-binding proteins: evidence that pregnancy-associated plasma protein A contributes to proteolysis of IGF-binding protein 5 in bovine follicular fluid. Endocrinology.

[39] Cataldo NA, Giudice LC (1992). Insulin-like growth factor binding protein profiles in human ovarian follicular fluid correlate with follicular functional status. J Clin Endocrinol Metab.

[40] de la Sota RL, Simmen FA, Diaz T, Thatcher WW (1996). Insulin-like growth factor system in bovine first-wave dominant and subordinate follicles. Biol Reprod.

[41] Stewart RE, Spicer LJ, Hamilton TD, Keefer BE, Dawson LJ, Morgan GL, Echternkamp SE (1996). Levels of insulin-like growth factor (IGF) binding proteins, luteinizing hormone and IGF-I receptors, and steroids in dominant follicles during the first follicular wave in cattle exhibiting regular estrous cycles. Endocrinology.

[42] Mihm M, Good TE, Ireland JL, Ireland JJ, Knight PG, Roche JF (1997). Decline in serum follicle-stimulating hormone concentrations alters key intrafollicular growth factors involved in selection of the dominant follicle in heifers. Biol Reprod.

[43] Bayasula, Iwase A, Kobayashi H, Goto M, Nakahara T, Nakamura T, Kondo M, Nagatomo Y, Kotani T, Kikkawa F: A proteomic analysis of human follicular fluid: comparison between fertilized oocytes and non-fertilized oocytes in the same patient. J Assist Reprod Genet. 2013, 30:1231–1238. 10.1007/s10815-013-0004-3 PMC3800524 23888310

[44] Liu YX, Ny T, Sarkar D, Loskutoff D, Hsueh AJ (1986). Identification and regulation of tissue plasminogen activator activity in rat cumulus-oocyte complexes. Endocrinology.

[45] Liu YX (2004). Plasminogen activator/plasminogen activator inhibitors in ovarian physiology. Front Biosci.

[46] Dow MP, Bakke LJ, Cassar CA, Peters MW, Pursley JR, Smith GW (2002). Gonadotropin surge-induced up-regulation of the plasminogen activators (tissue plasminogen activator and urokinase plasminogen activator) and the urokinase plasminogen activator receptor within bovine periovulatory follicular and luteal tissue. Biol Reprod.

[47] Smokovitis A, Kokolis N, Taitzoglou I, Rekkas C (1992). Plasminogen activator: the identification of an additional proteinase at the outer acrosomal membrane of human and boar spermatozoa. Int J Fertil.

[48] Huarte J, Vassalli JD, Belin D, Sakkas D (1993). Involvement of the plasminogen activator/plasmin proteolytic cascade in fertilization. Dev Biol.

[49] Ny T, Wahlberg P, Brandstrom IJ (2002). Matrix remodeling in the ovary: regulation and functional role of the plasminogen activator and matrix metalloproteinase systems. Mol Cell Endocrinol.

[50] Robker RL, Russell DL, Espey LL, Lydon JP, O’Malley BW, Richards JS (2000). Progesterone-regulated genes in the ovulation process: ADAMTS-1 and cathepsin L proteases. Proc Natl Acad Sci U S A.

[51] Tsafriri A, Bicsak TA, Cajander SB, Ny T, Hsueh AJ (1989). Suppression of ovulation rate by antibodies to tissue-type plasminogen activator and alpha 2-antiplasmin. Endocrinology.

[52] Murdoch WJ (1998). Regulation of collagenolysis and cell death by plasmin within the formative stigma of preovulatory ovine follicles. J Reprod Fertil.

[53] Perricone R, Pasetto N, De Carolis C, Vaquero E, Piccione E, Baschieri L, Fontana L (1992). Functionally active complement is present in human ovarian follicular fluid and can be activated by seminal plasma. Clin Exp Immunol.

[54] Georgiou AS, Gil MA, Alminana C, Cuello C, Vazquez JM, Roca J, Martinez EA, Fazeli A (2011). Effects of complement component 3 derivatives on pig oocyte maturation, fertilization and early embryo development in vitro. Reprod Domest Anim.

[55] Chen L, Mao SJ, McLean LR, Powers RW, Larsen WJ (1994). Proteins of the inter-alpha-trypsin inhibitor family stabilize the cumulus extracellular matrix through their direct binding with hyaluronic acid. J Biol Chem.

[56] Powers RW, Chen L, Russell PT, Larsen WJ (1995). Gonadotropin-stimulated regulation of blood-follicle barrier is mediated by nitric oxide. Am J Physiol.

[57] Hess KA, Chen L, Larsen WJ (1998). The ovarian blood follicle barrier is both charge- and size-selective in mice. Biol Reprod.

[58] Zhuo L, Yoneda M, Zhao M, Yingsung W, Yoshida N, Kitagawa Y, Kawamura K, Suzuki T, Kimata K (2001). Defect in SHAP-hyaluronan complex causes severe female infertility. A study by inactivation of the bikunin gene in mice. J Biol Chem.

[59] Fulop C, Szanto S, Mukhopadhyay D, Bardos T, Kamath RV, Rugg MS, Day AJ, Salustri A, Hascall VC, Glant TT, Mikecz K (2003). Impaired cumulus mucification and female sterility in tumor necrosis factor-induced protein-6 deficient mice. Development.

[60] Huang L, Yoneda M, Kimata K (1993). A serum-derived hyaluronan-associated protein (SHAP) is the heavy chain of the inter alpha-trypsin inhibitor. J Biol Chem.

[61] Vizcaino JA, Deutsch EW, Wang R, Csordas A, Reisinger F, Rios D, Dianes JA, Sun Z, Farrah T, Bandeira N (2014). ProteomeXchange provides globally coordinated proteomics data submission and dissemination. Nat Biotechnol.

