# Supplementary figures and images for: Follicular fluid biomarkers for human in vitro fertilization outcome: Proof of principle
Source: Proteome Sci. 2016 Nov 14;14:17. doi: 10.1186/s12953-016-0106-9 (PMC5109724; doi:10.1186/s12953-016-0106-9)

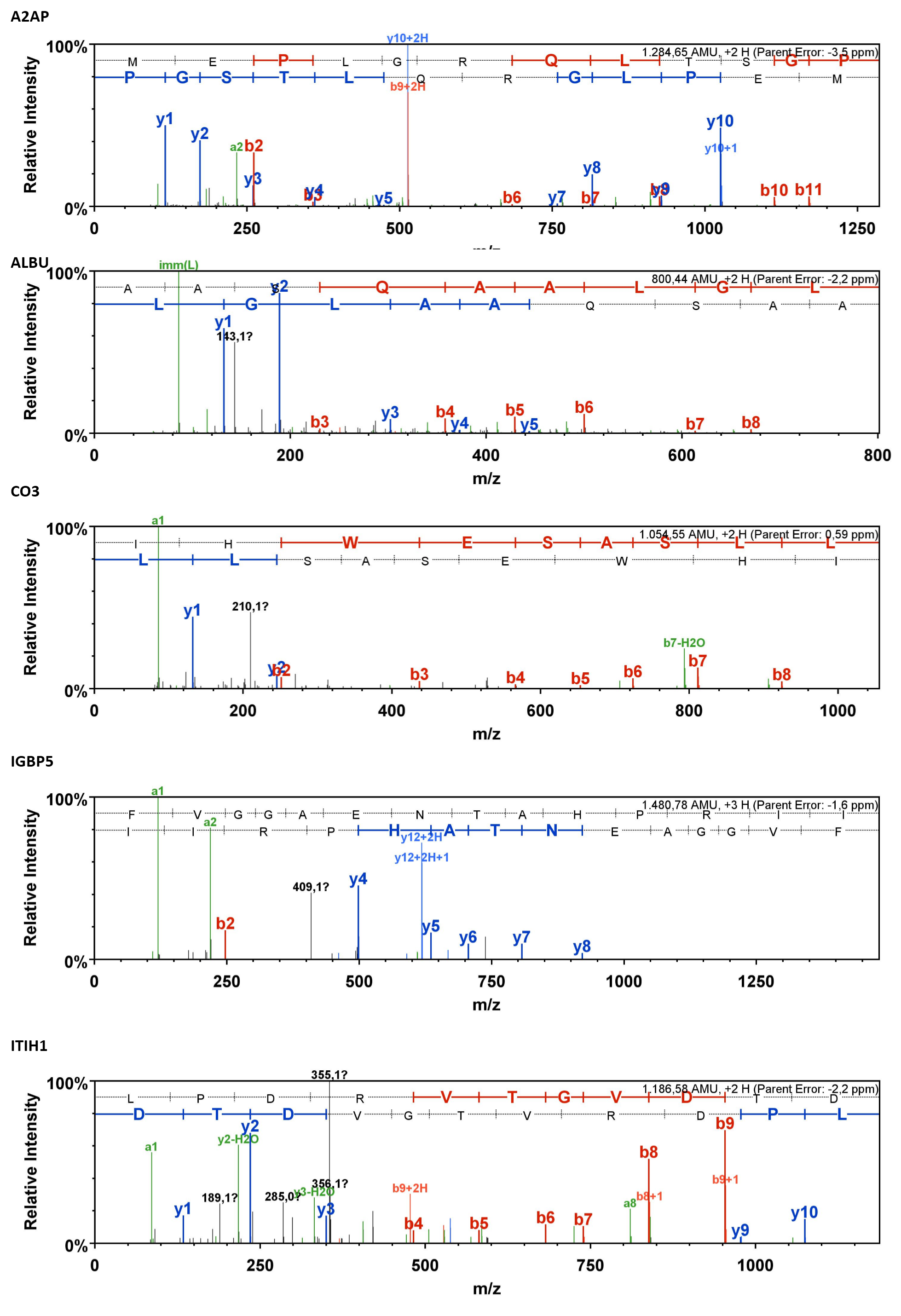

Supplement: Additional file 4: Figure S1. — MS spectrum form of identified peptides. (TIF 2099 kb) [file 12953_2016_106_MOESM4_ESM.tif]
